# A Comparative Study of Decoders for Liver and Tumor Segmentation Using a Self-ONN-Based Cascaded Framework

**DOI:** 10.3390/diagnostics14232761

**Published:** 2024-12-08

**Authors:** Sidra Gul, Muhammad Salman Khan, Md Sakib Abrar Hossain, Muhammad E. H. Chowdhury, Md. Shaheenur Islam Sumon

**Affiliations:** 1 Department of Computer Systems Engineering, University of Engineering and Technology, Peshawar 25000, Pakistan; sidragul.cse@uetpeshawar.edu.pk; 2 Artificial Intelligence in Healthcare, Intelligent Information Processing Lab, National Center of Artificial Intelligence, Peshawar 25000, Pakistan; 3 Department of Electrical Engineering, College of Engineering, Qatar University, Doha 2713, Qatar; sakib.hossain01@northsouth.edu (M.S.A.H.); mchowdhury@qu.edu.qa (M.E.H.C.); sumon3455.ms@gmail.com (M.S.I.S.)

**Keywords:** computer-aided diagnosis, medical image processing, liver segmentation, self-organizing neural networks, deep learning, convolution neural networks, tumor segmentation

## Abstract

**Background/Objectives:** Accurate liver and tumor detection and segmentation are crucial in diagnosis of early-stage liver malignancies. As opposed to manual interpretation, which is a difficult and time-consuming process, accurate tumor detection using a computer-aided diagnosis system can save both time and human efforts. **Methods:** We propose a cascaded encoder–decoder technique based on self-organized neural networks, which is a recent variant of operational neural networks (ONNs), for accurate segmentation and identification of liver tumors. The first encoder–decoder CNN segments the liver. For generating the liver region of interest, the segmented liver mask is placed over the input computed tomography (CT) image and then fed to the second Self-ONN model for tumor segmentation. For further investigation the other three distinct encoder–decoder architectures U-Net, feature pyramid networks (FPNs), and U-Net++, have also been investigated by altering the backbone at the encoders utilizing ResNet and DenseNet variants for transfer learning. **Results:** For the liver segmentation task, Self-ONN with a ResNet18 backbone has achieved a dice similarity coefficient score of 98.182% and an intersection over union of 97.436%. Tumor segmentation with Self-ONN with the DenseNet201 encoder resulted in an outstanding DSC of 92.836% and IoU of 91.748%. **Conclusions:** The suggested method is capable of precisely locating liver tumors of various sizes and shapes, including tiny infection patches that were said to be challenging to find in earlier research.

## 1. Introduction

Liver tumor segmentation is currently one of the most active research areas in medical image processing. Liver cancer has a high mortality rate, ranking sixth, and accounts for 10.2% of male deaths and 5.6% of female deaths worldwide [1]. The primary factors contributing to liver cancer are chronic liver disease infections, such as hepatitis B and C, alcohol consumption, diabetes, obesity, and chemical exposure. Liver tumors can have either a benign or malignant character. The liver is commonly affected by metastatic disease, with the colon, breast, lung, pancreas, and stomach being the principal sites of origin [2]. Due to the variety in the dimensions, morphology, consistency, and quantity of these tumors, precisely detecting them becomes a challenging endeavor. Segmenting a liver tumor is challenging due to both its size and the close proximity of intensity values between the tumor and the liver.

The primary imaging modalities utilized for the identification, diagnosis, and monitoring of liver tumors are computed tomography (CT), magnetic resonance imaging (MRI), ultrasound, and positron emission tomography (PET). Computed tomography (CT) is the most often utilized modality among several imaging techniques. The majority of manual tumor identification methods rely on a slice-by-slice analysis of 3D volumetric images. Due to the intricate nature of these procedures, they may be incorrect. Automatic systems, on the other hand, have become widely used in the past few years for the identification and treatment of liver diseases. These systems require nearly no human interaction and can detect liver diseases in a timelier manner. Automatic systems, on the other hand, have become widely used in the past few years for the identification and treatment of liver diseases.

Region- and shape-based approaches were prominent in the early days of liver tumor segmentation, while thresholding was also a popular option [3,4,5]. Then, for tumor segmentation from CT scan images, machine learning methods were developed [6,7]. Although machine learning techniques outperformed traditional segmentation approaches, these algorithms required a fundamental stage of feature engineering, which required a significant amount of human effort.

On the other hand, deep learning (DL) techniques do not require prior feature extraction, which has greatly contributed to their impressive achievements in several image processing fields. Convolutional neural networks (CNNs) and other deep learning algorithms are expected to be more efficient for various medical imaging diagnosis applications. In the absence of predetermined human criteria, a CNN has the potential to acquire unbiased and significant geographical characteristics through extensive training data. Consequently, a CNN has the potential to address the challenges related to tumor variability and feature design. U-Net [8], U-Net++ [9], ResNet [10], InceptionNet [11], MobileNetV1 [12], and attention-based CNNs [13] are popular deep learning designs that utilize the convolutional neural network (CNN) framework.

### 1.1. Related Work

CNN-based models have been widely used for segmentation tasks in recent decades. Ronneberger et al. [8] developed a CNN model for medical image segmentation called U-Net. Following the success of U-Net in computer vision applications, other variations of U-Net have been developed. A hybrid dense U-Net architecture proposed by Xiaomeng et al. [14] used two U-Nets densely connected to each other, where the first extracts the intra-slice features, and the other extracts the contextual information. The information from inter-slice and intra-slice features is then combined using the fusion algorithm. This approach resulted in a dice score of 96.5% for liver whereas a dice score of 82.4% in tumor segmentation. Jin et al. [15] created a U-Net based network called RA-UNet. The network presented in their work is a hybrid network and consists of attention-aware blocks incorporated in a 2D/3D U-Net architecture to merge both the low- and high-level features. The dice score for liver segmentation achieved was 96.3%, whereas the dice score for tumor segmentation was 79.5%.

Meng et al. [16] proposed a technique for segmenting tumors called a 3D convolutional neural network with dual scale. This network employed the dual-path methodology to perform segmentation and subsequently merged the outcomes from these routes. Conditional random fields have been employed in the post-processing stage for the aim of refining. The dice coefficient for tumor segmentation on the LiTS17 dataset was 0.689 using this approach.

Seo et al. [17] introduced a technique called modified U-Net (mU-Net). This strategy involves modifying the original U-Net architecture to avoid the replication of spatial and semantic information. The technique employed upsampling, which is tailored to each object and integrates high-level characteristics by utilizing skip connections in residual routes. This approach achieved a liver detection rate of 98.51% and a tumor detection rate of 89.72% using the LiTS17 dataset. Zhang et al. [18] introduced a network that utilizes the cascaded fully connected network (FCN) principle to segment liver and tumors in 3D images. A Dial-3DResUNet was developed specifically for liver segmentation, utilizing hybrid dilated convolution to effectively capture global features. On the other hand, a Hybrid-3DResUNet was constructed specifically for tumor segmentation. The model achieved a global dice score of 95.8% for liver segmentation and 74.2% for liver tumor segmentation on the 3DIRCADb dataset. In a study conducted by Budak et al. [19], a cascaded network was introduced. This network consisted of two ED-CNNs that were constructed and trained to detect liver areas and tumors in CT images. The initial ED-CNN partitions the liver image, producing a region of interest for the subsequent ED-CNN. The second ED-CNN subsequently partitions the tumor regions utilizing the regions of interest (ROIs) anticipated by the first ED-CNN. The constructed model was subsequently evaluated using 3DIRCADb, and a liver segmentation accuracy of 95.22% and a tumor segmentation accuracy of 64.3% were achieved using an openly available dataset.

Dong et al. [20] introduced a new hybrid network called the hybridized fully convolutional neural network (HFCNN) that was specifically designed for liver cancer segmentation. This approach included a combination of effective feature extraction techniques from the Inception model, together with the utilization of residual and pre-trained weights. This network attained an average dice coefficient of 92%.

Gao et al. [21] utilized a model that was based on a modified nested U-Net. In order to enhance the flow of gradients and maintain the integrity of features, the model used dilated dense short skip connections within the convolution blocks. The dice score for this model was 94.13%, whereas the SYSU-CT and subCT datasets achieved a score of 92.46%. Zhang et al. [22] proposed UV-Net, a novel 2.5D architecture that encodes information within the layer using 3D convolution and generates high-resolution outputs using 2D deconvolution. By implementing the suggested pre-processing technique of lowering mean energy, UV-Net demonstrated superior performance compared to previous methods in accurately segmenting the liver and enhancing the accuracy of segmenting small objects on the LiTS17 dataset. It reached an impressive dice coefficient of 88.92% for tumor segmentation.

Wang et al. [23] suggested a method that employs octave convolutions to learn multiple-spatial-frequency features, which allows it to capture tumors of various sizes and shapes more effectively. This U-Net encoder–decoder design included octave convolutions. The LiTS17 dataset was used to train this model and achieved a 96.3% dice score in delineating tumors. Lyu et al. [24] also proposed a new network named CouinaudNet that could estimate pseudotumor masks from Couinaud segment marks. The dataset called Medical Segmentation Decathlon Task08 comprised venous phase CT volumes that were used for evaluation purposes and a dice score of 69.5% was obtained for stage 1 and 74% for tumor segmentation. Tran et al. [25] presented another network constructed using U-Net and Un-Net. The outputs of the CNN blocks were used as the skip connections in the Un-Net model. On the LiTS17 dataset, a dice score of 96.38% for liver delineation was achieved and for the tumor the dice score is 73.69%.

Li et al. [26] introduced an advanced technique called an enhanced 3D feature pyramidal network (IFPN) to automatically partition stomach tumors that spread to other organs. The scope of this project was expanded to include liver tumor segmentation on the LiTS17 dataset, which yielded a dice score of 65.5%. Chi et al. [27] introduced an innovative framework comprising two components. The liver is dissected utilizing a complex network of branches, while its characteristics are organized in a pyramid-like form. The contextual information in the z-axis was likewise consolidated in 3D. The experimental findings were generated using the LiTS17 and 3DIRCADb datasets, achieving a segmentation accuracy of 96.68% for the liver and 69.11% for the tumor. Kushnure et al. [28] introduced a multi-scale approach to enhance the receptive field of a CNN-based network. The experimental findings on the 3Dircadb dataset yielded a dice similarity coefficient (DSC) of 97.13% for liver segmentation and 84.15% for tumor segmentation.

Wang et al. [29] constructed a network that is based on a U-Net architecture and incorporates squeeze and excitation (SE) and residual blocks. The SE block was employed for the recursive extraction of image characteristics, while atrous spatial pyramid pooling was utilized to change the transition and output layers. In addition, the typical convolution block is modified by incorporating residual structures in order to mitigate the problem of gradient vanishing. The performance of this technique was assessed on the LiTS 2017 and Sliver datasets, resulting in a dice score of 95.71% and 97.31%, respectively, for liver segmentation. Xu et al. [30] introduced a residual network called “PA-ResSeg” that incorporates a phase attention (PA) model. A PA-based multi-scale fusion (MSF) architecture was proposed to combine scale characteristics from multi-phase images. This architecture incorporates PA blocks at various stages along the encoding path of the network. The experiments utilized a multi-phase computed tomography imaging dataset that included focal malignancies. The achieved dice score was 86.82%.

Zhang et al. [31] introduced a network named DeepRecS that predicts the boundaries of liver tumors using a three-step method. A network for propagating response evaluation criteria in solid tumor (RECIST) marks was developed to predict similar markings according to RECIST. The study utilized a CT dataset comprising information from 231 patients diagnosed with hepatocellular carcinoma. The dataset was obtained from the First Affiliated Hospital of College of Medicine, Zhejiang University. The tumor segmentation achieved a dice score of 95.55%. Lei et al. [32] introduced a network called DefED-Net, which is capable of being deformed and consists of an encoder and a decoder. A dice value of 96.3% was reached for liver segmentation, while a dice coefficient of 87.52% was attained for tumor segmentation using the LiTS17 dataset.

### 1.2. Motivation and Novelty

Liver cancer ranks as the fourth most prevalent form of cancer globally [33]. Incidence of liver cancer is significantly higher in Sub-Saharan Africa and Southeast Asia compared to the United States. This form of cancer is the most common in several countries in these areas. Annually, a staggering number of over 800,000 individuals across the globe receive a diagnosis of this particular form of cancer, which ultimately leads to over 700,000 fatalities each year [34]. Hepatocellular carcinoma, often known as HCC, is caused by cirrhosis and the hepatitis B or C virus [35]. A CT scan is frequently employed for the diagnosis of liver cancer. Section 1.1 demonstrates extensive research on the segmentation of liver and liver cancers. While deep learning approaches have demonstrated effective liver segmentation, the segmentation of lesions still poses a hurdle. Manual tumor identification is prone to inaccuracies, leading to the misdiagnosis of liver malignancies, which can ultimately lead to fatal outcomes. Conversely, computer-assisted diagnosis systems necessitate minimal human involvement while also being time-efficient. The objective of this study is to create a system capable of accurately identifying the liver region in CT scans, distinguishing it from other organs, and identifying any cancers present within it.

This study made significant efforts to achieve this goal, combining deep pre-trained encoders with U-Net [9], U-Net++ [10], Self-ONN [36], and feature pyramid networks (FPNs) [37]. The encoder networks include ResNet18 [38], ResNet50, and ResNet152 [38] as well as different variations of DenseNet i.e., DenseNet121, DenseNet161, and DenseNet201 [39] were among the networks used in this study. U-Net++ with two backbones i.e., ResNet18 and ResNet50, has also been utilized. An innovative self-organized operational neural network (Self-ONN) model has been adopted at the decoder level to improve the segmentation of the liver as well as tumors in 3D CT images. The proposed design is a cascaded architecture that incorporates two independent networks. The liver is segmented by the first network, and the resulting liver mask is placed on the original CT images to form the liver region of interest (ROI). The ROI is then passed on to the second network, which segments the tumor. This study has the following novel contributions:

1. A cascaded strategy that uses two encoder–decoder networks one after the other has been created for segmenting liver and tumors, first for liver and then for tumor segmentation.
2. U-Net, FPN, and U-Net++ architectures along with novel Self-ONN models at the decoder level have been extensively trained not only for liver but also for tumor segmentation.
3. The concept of transfer learning has been utilized with different variations of DenseNet and ResNet pre-trained encoders as a backbone for different architectures, which significantly improves segmentation performance.
4. The superimposition of the segmented liver as a region of interest (ROI) from the first encoder–decoder network, before creating tumor segmentation by the second encoder–decoder network, has improved the overall performance of tumor segmentation.

The paper is constructed as follows: Section 2 explains the general methodology, including the dataset description, the segmentation algorithms used for the liver and tumor detection, experimental setup, resources utilized for this research, and the measurements employed to evaluate the performance of the model. The results are presented and analyzed in Section 3, and the work is concluded in Section 4.

## 2. Materials and Methods

**Figure 1:**
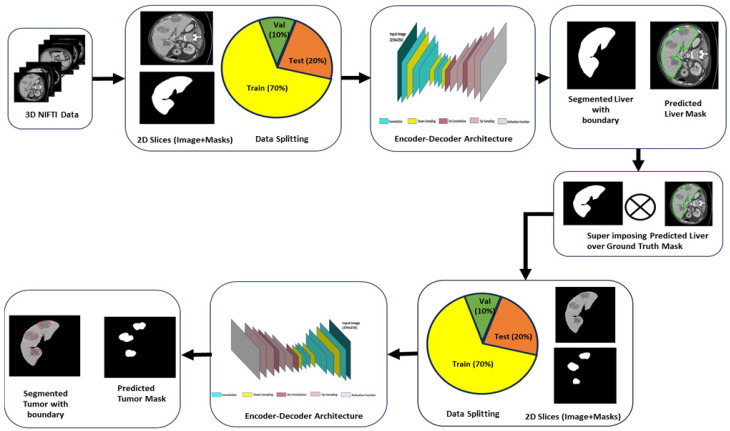
Flowchart of the proposed methodology.

The overall methodology comprises three main stages. First, the CT data are pre-processed. The data are then fed into the segmentation models for the delineation of liver and tumor separately. Two encoder–decoder CNN architectures have been utilized for this purpose. The liver is segmented first using a CT image in a slice-by-slice way using an encoder–decoder CNN architecture as shown in Figure 1.

The transfer learning mechanism has been applied to the encoder layer with ImageNet weights while training. The main purpose of transfer learning is to rapidly implement the models. The model will use the features it has learnt from other datasets that have finished the same task, rather than creating a dense neural network (DNN) from the start to solve the current problem. ResNet18 and DenseNet201 have been used for this purpose. The ImageNet dataset is used for pre-training these models.

### 2.1. Dataset Description

The current analysis [40] employs the LiTS17 benchmark dataset, which was also employed in the ISBI 2017 and MICCAI 2017 liver tumor segmentation (LiTS) challenges, which were conducted in 2017. In the Neuroimaging Informatics Technology Initiative (NIfTI) format, the dataset comprises 201 enhanced CT scans of the abdomen. Images have been divided into 70 and 131 volumes as training and test sets. Different scanners and protocols of various clinics have been used to collect this dataset due to which there is a large variation in the data quality, appearance, and voxel spacing. The training set has labeled data available for both liver and tumors, whereas for the test set the labeled data were not disclosed by the data supplier. The labeling of the data was performed by expert radiologists. These CT volumes consist of primary and secondary metastasis that has spread from the lungs. The quantity of CT slices ranges from 42 to 1026. In this study, 58,638 CT slices were employed for training, testing, and validation in the training dataset, utilizing five-fold cross-validation.

### 2.2. Data Pre-Processing

As described earlier, the LiTS17 dataset consists of three-dimensional images in NIFTI format. To process these data using our proposed methodology, these 3D CT data have been converted into 2D slices.

**Figure 2:**
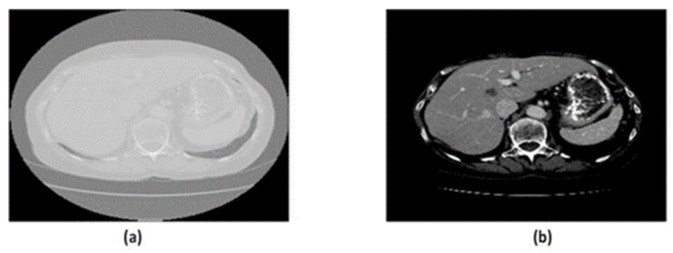
(**a**) Unprocessed Raw Image. (**b**) The image has been preprocessed using the Hounsfield unit.

The intensity values for the NIfTI format range between −1000 and 2000, which is invisible to the human eye. Therefore, the intensity values are normalized using Hounsfield unit (HU) windowing in the range of [−100,400] for the liver. Also, a range of 0–255 is used for intensity value mapping. Sample images have been shown in Figure 2.

### 2.3. Segmentation Models

The model design used in this study was adapted from the U-Net architecture, which has demonstrated remarkable success in biomedical picture segmentation. The U-Net model has a symmetrical structure, where on the left side it has a decoder that handles expansion and an encoder path that handles contraction. A rectified linear unit (ReLU) and a max-pooling operation follow each convolutional layer in the encoder segment. The network is capable of perceiving and understanding the global information contained within the image by employing a bottleneck, a condensed region that follows the encoder and contains additional convolutional layers. After the bottleneck phase, the decoder route starts the process of enhancing the feature maps and gradually bringing them back to their original spatial dimensions. The use of skip connections is one of the main features of U-Net. The decoding procedure involves the concatenation of feature maps from each encoder level to the corresponding levels in the decoder with these linkages. Effectively converting the multi-channel feature map into the final segmentation output, the 1 × 1 convolutional layer is the final architectural component. This outcome identifies the regions of interest in the image that are input.

By adopting the U-Net architecture, two advanced encoders have been utilized in our experiments, ResNet18 and DenseNet201. At the decoder side, Self-ONN blocks have been employed, replacing the conventional convolution layers in an effective manner. We substituted the conventional ReLU activation with a tanh activation function after each Self-ONN block.

#### 2.3.1. ResNet18

ResNet comes in a variety of models, including ResNet18, ResNet34, and ResNet50. The architecture remains the same and the numbers represent layers.

ResNets differ primarily in that they have shortcut connections that run parallel to their standard convolutional layers. Unlike convolution layers, these shortcut connections are always active, and gradients can easily backpropagate via them, resulting in speedier training. ResNets differ from standard ConvNets in a simple way. The idea is to create a clear path for gradients to propagate back to the network’s early levels. This accelerates the learning process by avoiding vanishing gradients.

#### 2.3.2. DenseNet201

In four benchmark tasks for object recognition: CIFAR-100, CIFAR-10, ImageNet, and SVHN, DenseNet [39] performs extremely well. A feed forward link is implemented in DenseNet’s architecture to facilitate the exchange of information among the network’s various layers. The vanishing gradient problem is addressed, feature reuse is increased, and the number of parameters is considerably reduced by DenseNet. Additionally, it offers several other benefits. DenseNet201’s designation is derived from its architecture, which comprises 201 layers. Exceptional performance can be achieved with minimal computational and memory requirements by this model. In this investigation, the final fully connected layer is abolished and replaced with the planned classifier, which incorporates the flatten layer, dense layer, and batch normalization layer.

#### 2.3.3. Self-ONN Architecture

Recent research on computer vision difficulties has shown that Self-ONN is remarkably effective [41,42,43,44]. We use a sophisticated neural network design called Self-ONN in the modification section, which overcomes the drawbacks of traditional CNNs, which rely on a single linear neuron model [45]. ONNs, which provide a more generalized neuron model that may be used in heterogeneous ONNs, were created as a solution to these issues. One major drawback of conventional ONNs is the computational difficulty of the operator search process, which results in the constraint that all neurons within a particular layer can only employ a single operator. The network’s ability to exhibit neuronal diversity is restricted by this limitation. To overcome this restriction, generative neurons are combined into a Self-ONN [35]. During the training phase, this technique allows the network to dynamically adjust and optimize the nodal operator for each connection. Any non-linear function’s value at a certain place can be roughly estimated using the qth order Taylor approximation. To boost performance and add uniqueness to the architecture, we introduce a new element to the bottleneck region of our proposed model by modifying it utilizing Self-ONN.

The Self-ONN ResBlock and Self-ONN Conv ResBlock take a n × n input and process it through a 3 × 3 Self-ONN layer in the Self-ONN Conv block to produce a 4n × 4n output. The output is subsequently passed through a 1 × 1 Self-ONN layer to preserve its 4n × 4n dimensions. The original n × n input is subsequently processed through a 3 × 3 convolutional layer, yielding a 4n × 4n output. Finally, the outputs of the 3 × 3 convolutional layer and the 1 × 1 Self-ONN layer are concatenated to provide a final output of size 4n × 4n.

The Self-ONN ResBlock block processes an input of size n × n via a 3 × 3 Self-ONN layer, resulting in an output of size n/4 × n/4. The output is then sent through a 1 × 1 Self-ONN layer, which reduces it to its original size of n × n. This output feature is then integrated with the first input feature using a residual link to produce a final output of size n × n.

The equation for the Taylor series function f(x) near point x = b_0_ is as follows:

(1) $$f\left(x\right)=f\left(b_{0}\right)+\frac{f^{\prime }\left(b_{0}\right)}{1\;!}\left(x-b_{0}\right)+\frac{f^{\prime \prime }\left(b_{0}\right)}{2\;!}{\left(x-b_{0}\right)}^{2}+\cdots \frac{f^{q}\left(b_{0}\right)}{q!}{\left(x-b_{0}\right)}^{q}$$

(2) $$f\left(x\right)=f\left(b_{0}\right)+\frac{f^{\prime }\left(b_{0}\right)}{1\;!}\left(x\right)+\frac{f^{\prime \prime }(b_{0})}{2\;!}{\left(x\right)}^{2}+\cdots \frac{f^{q}\left(b_{0}\right)}{q!}{\left(x\right)}^{q}$$

(3) $$f\left(x\right)=c+v_{1}\left(x\right)+v_{2}{\left(x\right)}^{2}+\cdots +v_{q}\;{\left(x\right)}^{q}$$

The backpropagation procedure optimizes the coefficient values $v_{1}$, $v_{2}$,…, $v_{q}$ in Equation (3). Any ONN operation can be formulated as

(4) xr^ka,b=Pnk (ψrkwrka,b,yr−1a−u,b−vu,v=(0,0)(p−1,q−1)

In Equation (4) $P_{r}^{k}\left(.\right):R^{MN\times K^{2}}\to R^{MN}$ in the pool operator, and $\psi_{r}^{k}\left(.\right):R^{MN\times K^{2}}\to R^{MN\times K^{2}}$ in the nodal operator.

#### 2.3.4. Proposed Network

The liver is segmented using the first network within a CT slice by constructing a binary mask, which assigns 1 to liver pixels and 0 to the ground truth pixels. Then, the binary mask for the liver is concatenated with the input CT image to extract the region of interest, which in our case is the liver. Then, using the second network, the tumor is segmented by feeding the ROI generated from the first step. This section covers the dataset utilized in the experiment, as well as the pre-processing techniques that were performed on the dataset and the networks that were utilized to segment the liver and tumor from volumetric images.

Two distinct networks were utilized to segment both the liver and lesions from CT images. The first network generates a hepatic mask by processing 2D CT slices, while the second deep learning model segments tumors using the previously segmented liver region. Both models leverage encoder–decoder convolutional neural network architectures for segmentation tasks. Specifically, the networks employ advanced U-Net and feature pyramid network (FPN) architectures, incorporating DenseNet and ResNet variants as encoder backbones to enhance performance.

ResNet with 18, 50, and 152 layers as well as DenseNet with 121, 161, and 201 layers have been used as backbone models. The encoder–decoder architectures that have been employed for segmentation are able to capture the contextual information in the contracting path which is helpful in the localization of the liver and tumor in the expanding path. In the U-Net design, the output from the last decoding layer is mapped into two-channel feature maps using a 1 × 1 convolution. For each model of liver and tumor, a SoftMax activation function is applied for segmenting the pixels into the liver or tumor class and background class.

**Figure 3:**
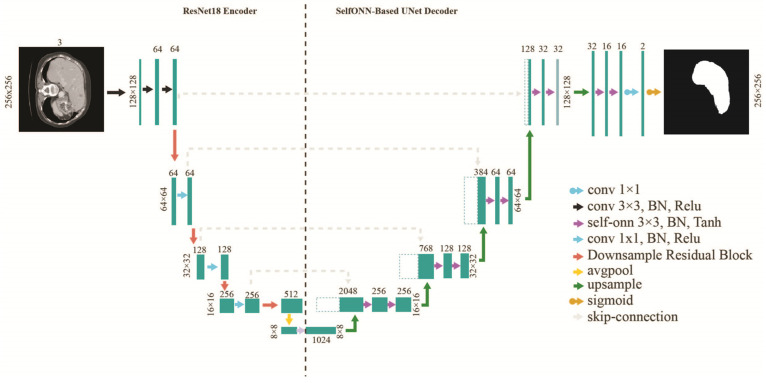
Network Architecture for liver segmentation.

**Figure 4:**
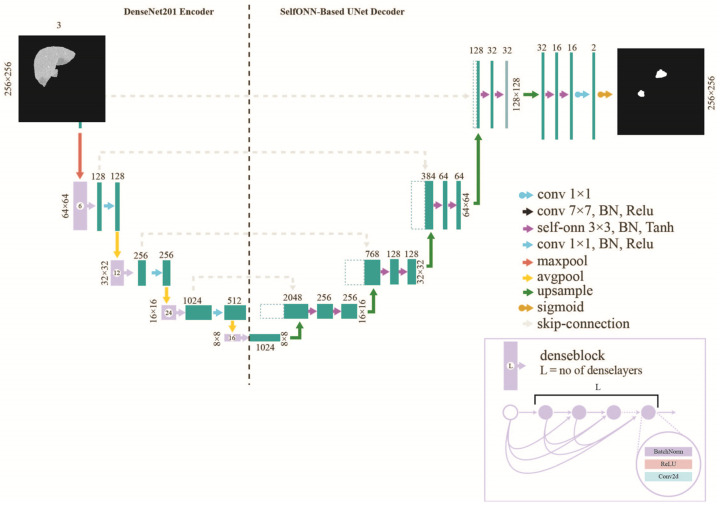
Network Architecture for tumor segmentation.

The FPN architecture consists of an encoder–decoder as a pyramid structure in which the predicted feature maps are created at spatial levels of the decoder. The predicted feature maps are then upsampled to input size and concatenated and convolved with a 3 × 3 kernel and finally pass through the SoftMax activation function for the outcome. The convolutional layers at the encoder side are initialized with the ImageNet weights. During training, the encoder layer was trained using the transfer learning mechanism with ImageNet weights. The architecture of the liver and tumor segmentation models is also illustrated in Figure 3 and Figure 4.

### 2.4. Experimental Setup

The segmentation models were constructed on a system that was endowed with a 12-GB NVIDIA^®^ TITAN X(Pascal)^®^ GPU card, 16 GB of RAM, and an Intel^®^ Xeon^®^ CPU E3-1200/1500 v5 operating at 3.20 GHz. The PyTorch library was applied to the system. The segmentation models represented in Table 1 were trained with a learning rate of 10^−3^ using the Adam optimizer. Updates to momentum were implemented with values of 0.999 and 0.9. The training process consists of 40 backpropagation epochs and a collection size of 16 images. In the event that the validation loss did not ameliorate after five epochs, training was abruptly terminated in accordance with an early stopping criterion. The hyper-parameters and training data for the liver and tumor segmentation models are presented in Table 1.

When training, a 5-fold cross-validation method was employed, using 80% training data and 20% test data, to avoid overfitting. A 20% validation set was created from the training set. Counts of training, validation, and test photos are displayed in Table 2 for each fold. The liver segmentation models are fed by the original CT slices; the lesion segmentation network, which relies on tumor masks as a reference point, receives the CT slices that have been segmented from the liver. The suggested system’s performance is evaluated by combining the best models for tumor and liver segmentation.

### 2.5. Loss Function

In the above segmentation tasks, the dice loss is employed as a cost function, given by Equation (5) as:

(5) $$D=\frac{2\sum_{i}^{N}pigi}{\sum_{i}^{N}{p_{i}}^{2}+\sum_{i}^{N}{g_{i}}^{2}}$$

The predicted binary segmentation volume pi ∈ P, which corresponds to the predicted pixels, and the ground truth binary segmentation volume gi ∈ G, representing the ground truth pixels, are summed across *N* × *N* voxels.

### 2.6. Evaluation Criteria

The efficacy of liver and tumor segmentation was evaluated utilizing quantitative metrics. This evaluation employed pixel-by-pixel analysis, wherein pixels in the background were assigned to the negative class and pixels in the foreground were assigned to the positive class. A value of 1.96 was assigned to the 95% confidence intervals (CIs) utilized in the performance evaluation.

The efficacy of the liver and tumor segmentation networks was assessed using three metrics: correlation over union (IoU), dice coefficient (DSC), and accuracy. These are as follows:

(6) $$Accuracy=\frac{TP+TN}{TP+TN+FP+FN}$$

where the accuracy ratio is the number of successfully recognized pixels divided by image pixels. True positive and true negative are TP and TN in Equation (3), while false positive and false negative are FP and FN.

(7) $$IntersectionoverUnion\left(IoU\right)=\frac{TP}{TP+FP+FN}$$

(8) $$Dice\;Similarity\;Coefficient\;\left(DSC\right)=\frac{2TP}{2TP+FP+FN}$$

where IoU and DSC measure the area that is spatially overlapped between the ground truth and the predicted segmentation masks. DSC and IoU are identical except for the fact that DSC gives TP pixels twice as much weight as IoU.

## 3. Results and Discussion

The segmentation results for both livers as well as tumor segmentation using the different networks with U-Net, Self-ONN, U-Net++, or FPN architecture with different encoders are discussed in Section 3.

### 3.1. Liver Segmentation

**Figure 5:**
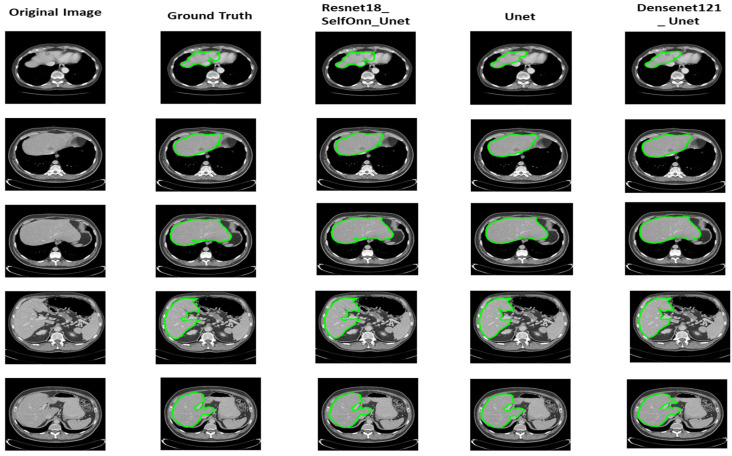
The columns from left to right show the comparison of different liver segmentation networks. The first column shows the original images, followed by the ground truth images. The outcomes of the top three performance networks with the highest dice scores are shown in the remaining three columns. The liver in the ground truth and the segmented images has been highlighted with a green boundary.

The quantitative results for liver segmentation have been given in Table 3 whereas Figure 5 shows the liver boundary segmented by the best three models with corresponding original and ground truth images. To segment the liver from volumetric CT images, different DenseNet and ResNet backbones were used in conjunction with U-Net, U-Net++, Self-ONN and FPN architectures. Among the results obtained for these networks, it is observed that the ResNet18_Self-ONN_U-Net, vanilla U-Net, and U-Net with DenseNet121 encoder performed the best, achieving dice similarity coefficients (DSCs) of 98.182%, 97.606%, and 97.344%, respectively.

The U-Net architecture, which used ResNet152 as an encoder, performed well as well, with a DSC of 97.344%. ResNet18 and ResNet50, which were used as encoders with U-Net, achieved almost similar results with DSCs of 97.24% and 97.256%, respectively. The minimum DSC for a U-Net network with a DenseNet161 encoder as a backbone is 97.01%. These results from the U-Net architecture with different backbones show that the U-Net network segmented the liver efficiently and produced results that were very close to the ground truth for the liver.

Although the FPNs with different encoders did not perform significantly better than the encoders with the U-Net network, they did not perform significantly worse, however, the FPN with the ResNet18 encoder achieved an average DSC of 97.26%. In addition, the DSC of the DenseNet161 encoder with the FPN was 97.09%, which is higher than the DSC of the DenseNet161 encoder with the U-Net network, i.e., 97.01%.

Based on the above performance of the ResNet18 and ResNet50 encoders, another architecture, U-Net++, was used as the backbone encoder with these two encoders. U-Net++ with the ResNet18 encoder produced excellent results with a DSC of 97.514 percent, whereas the ResNet50 encoder produced results that were not comparable to those of ResNet18.

As can be seen from Table 3, the vanilla U-Net network also achieved remarkable performance with a dice similarity coefficient (DSC) of 97.606%. It can be observed from the qualitative assessment that the liver mask produced by ResNet18_Self-ONN_U-Net, U-Net, and DenseNet121 with U-Net is highly consistent with the ground truth. The first column of Figure 4 displays the original CT slice, the second column contains the ground truth mask, and the third, fourth, and fifth columns have the predicted masks from the three most effective models for liver segmentation. The ground truth as well as predicted masks have overlapped over original images, which is further highlighted with a green boundary in order to show more clarity. It can be seen from the segmentation masks that the vanilla U-Net network has produced segmentation masks, even for a small region of the liver. U-Net with a DenseNet121 encoder and U-Net++ with a ResNet18 encoder, the other two high-performance networks, have also segmented the liver into small areas, achieving the highest level of accuracy.

### 3.2. Tumor Segmentation

Table 4 summarizes the results of tumor segmentation using the LiTS dataset. The Self-ONN–U-Net model with a DenseNet201 backbone has performed the best for tumor segmentation and achieved a dice similarity coefficient of 92.872%. The second-best model is U-Net++ with a ResNet50 backbone which achieved a DSC of 92.836%. The third-best network is U-Net with a DenseNet201 backbone and obtained a dice similarity coefficient of 92.828%. Table 4 shows that the U-Net network with various backbone encoders outperformed the FPN with various backbone encoders similar to those used in liver segmentation networks. U-Net with DenseNet161 and DenseNet121 as a backbone are other networks that achieved DSCs of 92.69% and 92.618%, respectively. The DSC of the ResNet152 encoder with U-Net, on the other hand, was 91.762%, which is higher than the DSC of the ResNet18 and ResNet50 encoders with U-Net, achieving 91.748% and 91.714%, respectively. On the other hand, FPNs with different combinations did not perform well and achieved a 3% lower dice score than the U-Net networks. The maximum DSC achieved by the FPN is 89.628%, with the DenseNet161 encoder as the backbone. As a result, the FPN is not the best choice for segmenting liver tumors from 3D volumetric images.

Similar to liver segmentation, U-Net++ with ResNet18 and ResNet50 encoders have also been used for tumor segmentation. U-Net++ with ResNet50 has proved to be the best segmentation network, whereas the ResNet18 encoder with U-Net++ comparatively did not perform well.

**Figure 6:**
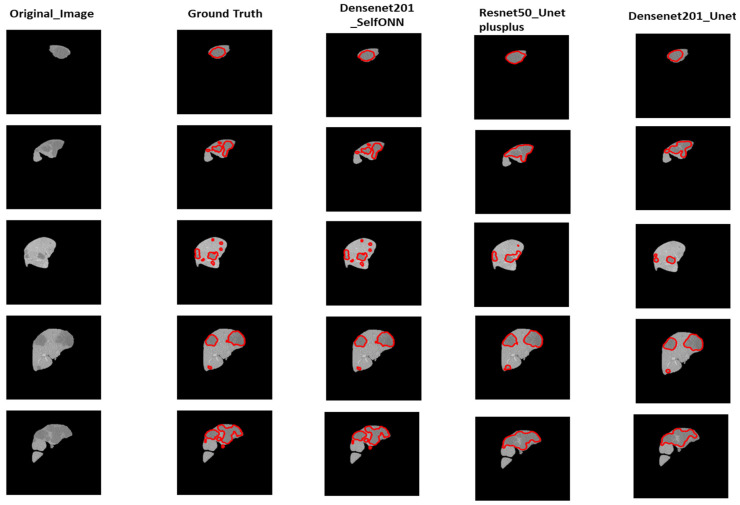
Performance comparison of different tumor segmentation networks(the red boundary shows the predicted pixels).

The results of tumor segmentation for the three best models have been shown in Figure 6 with the original liver and ground truth lesion mask annotated by medical experts. It can be seen from the results that U-Net++ has segmented small tumors within the liver as compared to the other two best-performing networks that have missed some of the tumors to be segmented.

Table 5 shows the number of images achieving a dice score of zero. As can be noticed from the above data, these 0% dice scores, obtained owing to incorrect segmentation, contribute to false positives and false negatives, hence affecting the overall performance of the system.

In our work, the performance variations among decoder architectures result from their different designs and the particular needs of liver and tumor segmentation tasks. High DSC and IoU scores follow from U-Net’s [8] symmetric encoder–decoder structure with skip connections improving localization accuracy. ResNet-based U-Nets [46] allow deeper networks by introducing residual connections to solve the vanishing gradient problem; yet, our results imply that, particularly with limited data, increasing depth does not always improve performance and may cause overfitting. DenseNet-based U-Nets [47] encourage feature reuse via dense connections; DenseNet121_U-Net strikes a balance between computational economy and performance. Although they manage multi-scale features, FPNs [48] did not outperform other models in our tests, maybe because of the particular quality of the medical images used. Although U-Net++ [9] presents stacked and thick skip paths to capture fine-grained features, it did not routinely outperform the original U-Net in our application. We included self-organized operational neural networks (Self-ONNs) [35] in the decoder of our model to solve the limits of conventional convolutional layers, which depend on linear neuron models. Self-ONNs improve the capacity of the network to learn difficult functions by providing a generalized neuron model able to represent several non-linear operators. Although they include non-linear operators, traditional operational neural networks (ONNs) suffer with low neuronal diversity resulting from the usage of a single operator per layer and computational complexity in operator search. Using the qth order Taylor approximation to approximate non-linear functions, Self-ONNs address these issues by incorporating generative neurons that dynamically alter and optimize the nodal operator for every connection during training. By better capturing intricate patterns and structures inside the data, including Self-ONNs in the bottleneck area increases the representational capacity and flexibility of the model, hence improving segmentation performance. All things considered, the intrinsic design concepts of every decoder architecture and the particular needs of liver and tumor segmentation tasks define the observed performance variations. By adding dynamic and flexible non-linear operations, which increases segmentation accuracy and helps the network to describe complicated relationships inside the data, Self-ONNs solve the limits of conventional CNNs.

**Figure 7:**
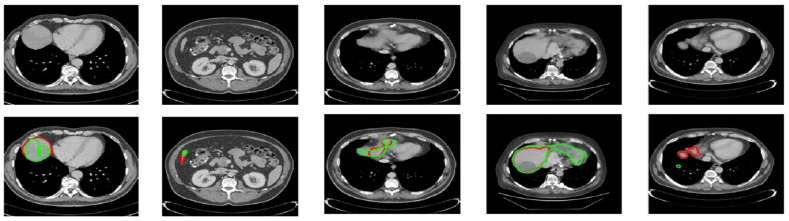
Original images are shown in the first row. The second row shows the boundaries of the ground truth and predicted liver images laid over each other. The green boundary shows the ground truth while the red boundary shows the predicted pixels.

**Figure 8:**
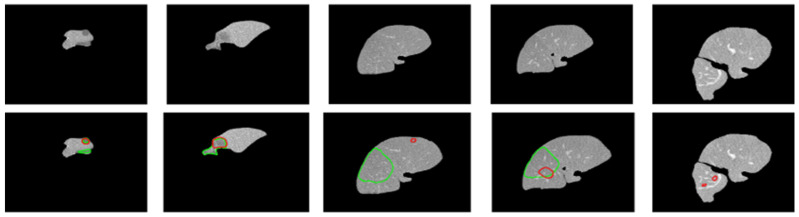
The first row shows the original images. The second row shows the boundaries of the ground truth and predicted tumor images laid over each other. The green boundary shows the ground truth pixels whereas the red boundary shows the predicted pixels.

Figure 6 shows the performance comparison of different tumor segmentation networks, where the red boundary represents the predicted pixels. Figure 7 and Figure 8 show the mask overlay of ground truth and the predicted mask of the liver and tumor for the samples. The second rows in Figure 7 and Figure 8 show the regions highlighted with red and green boundaries. These borders indicate the incorrect interpretations of the models in the case of liver and tumor prediction. The green boundaries indicate the actual liver and tumor locations in the image, while the red boundaries highlight the model-predicted liver and tumor regions. As a result, this misinterpretation of the models, which predicted the incorrect liver and tumor locations, did not contribute to the dice score, affecting the overall dice score for both the liver and tumor.

Table 6 shows the performance comparison of our network with other cascaded liver and tumor networks. As can be seen, our network has performed better than the other state-of-the-art networks.

### 3.3. Model Complexity Analysis

The performance, efficiency, and fit for various tasks of a model depend much on its complexity. Especially in real-time or resource-limited contexts, optimal results in segmentation tasks depend on a compromise between model accuracy and processing resources. The complexity analysis of two segmentation models, ResNet18_Self-ONN_U-Net and DenseNet201_Self-ONN_U-Net, is presented in Table 7. The ResNet18-based model is comparatively lightweight, as it has a total of 20,622,065 trainable parameters. This reduced number of parameters suggests a reduced computational overhead, which can be advantageous in live segmentation applications where resource efficiency and performance are critical. Conversely, the DenseNet201-based model, which comprises 49,546,289 parameters, is computationally more intensive. The increased intricacy of the system enables it to capture more complex patterns, which could potentially enhance its performance in tasks such as tumor segmentation, where detailed feature extraction is required. The Self-ONN framework is integrated with U-Net in both models, which enhances the accuracy of segmentation by learning hierarchical features across multiple dimensions.

The inference times for two segmentation models, ResNet18_Self-ONN_U-Net and DenseNet201_Self-ONN_U-Net, are reported in Table 8. The ResNet18-based model has a substantially shorter total inference time for processing patient data with 300 slices on average, with a total time of 2.17 s, as opposed to the DenseNet201-based model, which has a total timing of 10.54 s. This discrepancy is indicative of the difference in model complexity, as the ResNet18 model is less computationally intensive, thereby enabling quicker inference. The DenseNet201 model processes each image in 0.035126 s, while the ResNet18 model processes each image in 0.007244 s when considered on a per-image basis. These discrepancies emphasize the trade-off between model accuracy and inference speed, with ResNet18 potentially sacrificing some accuracy due to its simpler architecture, despite its superior efficiency for real-time applications. In contrast, DenseNet201, despite its decreased processing speed, may provide superior performance in terms of feature extraction and segmentation accuracy, rendering it more appropriate for tasks that necessitate a higher level of precision, such as tumor segmentation.

## 4. Conclusions

In this paper, we employed a variety of encoders, including ResNet18, ResNet50, ResNet152, DenseNet121, DenseNet161, and DenseNet201, in conjunction with U-Net, FPN, Self-ONN, and U-Net++ network architectures, to attain efficient liver and tumor segmentation from CT images. It can be shown that Self-ONN with ResNet18 and Self-ONN with DenseNet201 have achieved the best performances for liver and tumor, respectively. For liver segmentation, ResNet18_Self-ONN_U-Net performed well, while for tumor segmentation, DenseNet201_Self-ONN_U-Net performed better compared to the other models employed at the encoder and decoder sides. In contrast, the FPN was unable to surpass the U-Net network in the category of liver and tumor segmentation. Although the results for liver segmentation among different 2D CNN models are not significantly different, the results for tumor segmentation are quite different. Furthermore, the 3D to 2D conversion is a time-consuming process that necessitates computations.

This work could also be extended to 3D segmentation to restore both spatial and contextual information. The deep learning architecture of the proposed study can be leveraged to create applications like computer-aided diagnostic (CAD) systems. Because of the proposed model’s high accuracy, it can be compared to human accuracy for both liver and tumor segmentation. As a result, this work can be expanded to include the creation of CAD systems that automate the entire process, saving time and human effort.

## Figures and Tables

**Table 1:** Model parameters for liver and tumor segmentation training.

Training Parameters	Liver Segmentation	Tumor Segmentation
Learning rate	0.0001	0.0001
Epoch patience	6	6
Number of folds	5	5
Learning rate drop factor	0.2	0.2
Optimizer	Adam	Adam
Weights	ImageNet	ImageNet
Max epochs	50	50
Epoch stopping criteria	10	10
Batch size	8	8
Loss function	SMP dice	SMP dice loss

**Table 2:** Number of samples for training, testing, and validation.

Number of Samples	Liver Segmentation	Tumor Segmentation
Training	37,528	37,528
Testing	11,728	11,728
Validation	9382	9382

**Table 3:** The performances of the networks used for liver segmentation in terms of Accuracy, IoU, and DSC. The results of the three best networks have been highlighted.

Network	DSC (%)	IOU (%)	Accuracy (%)
**U-Net**	**97.606 ± 0.12**	**96.654 ± 0.15**	**99.872 ± 0.03**
ResNet18_U-Net	97.24 ± 0.13	96.152 ± 0.16	99.858 ± 0.03
ResNet50_U-Net	97.256 ± 0.13	96.262 ± 0.15	99.858 ± 0.03
ResNet101_U-Net	96.870 ± 0.22	95.674 ± 0.24	99.824 ± 0.01
ResNet152_U-Net	97.344 ± 0.13	96.34 ± 0.15	99.858 ± 0.05
**DenseNet121_U-Net**	**97.524 ± 0.13**	**96.552 ± 0.15**	**99.864 ± 0.03**
DenseNet161_U-Net	97.01 ± 0.14	95.846 ± 0.16	99.836 ± 0.03
DenseNet201_U-Net	97.108 ± 0.14	95.978 ± 0.16	99.842 ± 0.03
ResNet18_FPN	97.26 ± 0.13	96.158 ± 0.16	99.862 ± 0.03
ResNet50_FPN	96.908 ± 0.14	95.698 ± 0.16	99.836 ± 0.03
ResNet152_FPN	96.624 ± 0.15	95.386 ± 0.17	99.824 ± 0.03
DenseNet121_FPN	97.096 ± 0.14	95.926 ± 0.16	99.84 ± 0.03
DenseNet161_FPN	97.09 ± 0.14	95.928 ± 0.16	99.846 ± 0.03
DenseNet201_FPN	96.778 ± 0.14	95.552 ± 0.17	99.828 ± 0.03
ResNet18_U-Net++	97.514 ± 0.13	96.462 ± 0.15	99.866 ± 0.03
ResNet50_U-Net++	96.85 ± 0.14	95.698 ± 0.16	98.892 ± 0.08
**ResNet18_Self-ONN_U-Net**	**98.182 ± 0.10**	**97.436 ± 0.12**	**99.912 ± 0.02**

Note: Bold values indicate the best results among all the models.

**Table 4:** Accuracy, IoU, and DSC for tumor segmentation.

Network	Accuracy (%)	IOU (%)	DSC (%)
U-Net	99.932 ± 0.02	90.966 ± 0.23	92.03 ± 0.22
ResNet18_U-Net	99.934 ± 0.02	90.642 ± 0.24	91.748 ± 0.22
ResNet50_U-Net	99.908 ± 0.02	90.546 ± 0.24	91.714 ± 0.22
ResNet101_U-Net	99.932 ± 0.02	90.768 ± 0.91	91.802 ± 0.86
ResNet152_U-Net	99.872 ± 0.02	90.672 ± 0.24	91.762 ± 0.22
DenseNet121_U-Net	99.926 ± 0.02	91.626 ± 0.22	92.696 ± 0.21
DenseNet161_U-Net	99.93 ± 0.02	91.524 ± 0.23	92.618 ± 0.21
**DenseNet201_U-Net**	**99.938 ± 0.02**	**91.798 ± 0.22**	**92.828 ± 0.21**
ResNet18_FPN	99.878 ± 0.03	88.168 ± 0.26	88.168 ± 0.26
ResNet50_FPN	99.878 ± 0.03	88.168 ± 0.26	88.168 ± 0.26
ResNet152_FPN	99.878 ± 0.03	88.168 ± 0.26	88.168 ± 0.26
DenseNet121_FPN	99.86 ± 0.03	87.098 ± 0.27	87.344 ± 0.27
DenseNet161_FPN	99.892 ± 0.03	89.158 ± 0.25	89.628 ± 0.25
DenseNet201_FPN	99.874 ± 0.03	88.462 ± 0.26	88.646 ± 0.26
ResNet18_U-Net++	99.916 ± 0.02	88.734 ± 0.24	89.898 ± 0.26
**ResNet50_U-Net++**	**99.922 ± 0.02**	**91.748 ± 0.21**	**92.836 ± 0.22**
**DenseNet201_Self-ONN_U-Net**	**99.956 ± 0.02**	**91.768 ± 0.21**	**92.872 ± 0.10**

Note: Bold values indicate the best results among all the models.

**Table 5:** Number of samples achieving 0% dice score.

Experiments	Network	Total Samples	Samples with 0% Dice Score				
			Fold 1	Fold 2	Fold 3	Fold 4	Fold 5
Liver Segmentation	U-Net	11,728	150	156	154	128	149
	ResNet18_U-Net++	11,728	312	145	107	146	107
	DenseNet121_U-Net	11,728	167	159	182	175	131
Tumor Segmentation	ResNet50_U-Net++	11,728	556	630	550	598	621
	DenseNet121_U-Net	11,728	633	743	533	514	629
	DenseNet201_U-Net	11,728	541	580	603	614	772

**Table 6:** Comparison of different networks for liver and tumor segmentation in literature for the LiTS17 dataset.

Networks	Results	
	Liver Segmentation (DSC)	Tumor Segmentation (DSC)
[49] FED Net	-	76.6%
[15] RA-UNet	96.3%	79.5%
[17] Modified U-Net (mUNet)	98.51%	89.72%
[19] Cascaded Encoder–Decoder	95.22%	64.3%
[21] ASU-Net++	94.13%	92.46%
[28] Multi-scale U-Net (MS-UNet)	96.68%	69.11%
[32] Deformable Encoder–Decoder Network	96.3%	87.52%
Proposed Methodology	98.182%	92.872%

**Table 7:** Complexity Analysis of Models.

Models	Total Params	Trainable Params
**ResNet18_Self-ONN_U-Net**	20,622,065	20,622,065
**DenseNet201_Self-ONN_U-Net**	49,546,289	49,546,289

**Table 8:** Inference time of models.

	Per Patient on Average	Per Image
**ResNet18_Self-ONN_U-Net**	2.17 s	0.007244 s
**DenseNet201_Self-ONN_U-Net**	10.54 s	0.035126 s

## Data Availability

The dataset called liver tumor segmentation (LiTS) benchmark has been used for the training and evaluation of the model. https://academictorrents.com/details/27772adef6f563a1ecc0ae19a528b956e6c803ce (accessed on 7 December 2024).
